# Identification of Metagenome-Assembled Genomes Containing Antimicrobial Resistance Genes, Isolated from an Advanced Water Treatment Facility

**DOI:** 10.1128/MRA.00003-20

**Published:** 2020-04-02

**Authors:** Blake W. Stamps, John R. Spear

**Affiliations:** a Department of Civil and Environmental Engineering, Colorado School of Mines, Golden, Colorado, USA; Georgia Institute of Technology

## Abstract

Here, we present 95 metagenome-assembled genomes (MAGs) that harbor antimicrobial resistance genes, isolated from samples obtained in a large advanced wastewater reclamation facility prior to microfiltration. The MAGs were not in abundance after filtration at the facility and represent a useful resource to the water treatment community at large.

## ANNOUNCEMENT

Human society faces an acceleration of water scarcity due to increasing population, pollution, and land use. Such water scarcity also brings a decrease in water quality, as demonstrated by increased eutrophication, among other concerns (1). Treatment and reuse of water are critical tools in combating water stress globally. Reuse of wastewater carries the potential risk of transmission of small-molecule metabolites and antibiotic resistance genes (ARGs) (2), while its use relieves stress on natural sources of water. Previously, biofilms were sampled from the Orange County Water District (OCWD) Advanced Water Purification Facility (AWPF) in Southern California, which showed differences in the microbial communities in both influent and biofilms on microfiltration and reverse osmosis membranes (3). In a recent study that more fully characterized both biofilms and large volumes of water throughout the treatment process, we described both microbial diversity and load decreasing across this well-engineered system (4). Here, we have expanded and enhanced this previous work (4) by identifying metagenome-assembled genomes (MAGs) that contain ARGs to expand our knowledge of all microbial lineages harboring ARGs that are present within an ultrapurified water facility.

Water and biofilm were sampled at the OCWD AWPF as previously described (4). Briefly, all water samples were filtered using a large-volume concentrator (LVC) dialysis filter cartridge system (Innova Prep LLC, Drexel, MO). Approximately 60 to 100 liters of water were filtered per sample, prior to concentration onto replicate 25-mm (diameter), 0.22-μm (pore size) nitrocellulose filters. Filters were placed in BashingBead lysis tubes (Zymo, Inc., Irvine, CA) containing 750 μl of DNA/RNA Shield (Zymo, Inc.) to preserve samples onsite. Biofilm samples were similarly placed in lysis tubes with DNA/RNA Shield. DNA was extracted using the Zymo Microbiomics DNA/RNA coextraction kit (Zymo, Inc.). Libraries were prepared as previously described in full (4) using the Nextera XT library prep kit (Illumina, San Diego, CA), modified to use 13 cycles of PCR. All samples were sequenced on an Illumina HiSeq 3000 instrument using PE150 chemistry. Two biofilm samples from filtration units at the AWPF obtained from Leddy et al. (3) were processed as described previously and sequenced using SE100 chemistry. Sequence reads were error corrected using BayesHammer v3.12.0 (5) and assembled using MEGAHIT v1.1.3 (6) to produce a single coassembly. Reads were mapped to the assembly with Bowtie 2 v2.3.4.1 (7) and binned into MAGs using Anvi’o v v5 and CONCOCT (8, 9). MAG completion and redundancy estimates were also computed within Anvi’o. MAGs were queried for ARGs using DeepARG (10) and identified phylogenetically by GTDB-Tk v0.3.0 (11). Default parameters were used for all software unless otherwise noted.

A total of 95 MAGs were selected after manual curation within Anvi’o and by DeepARG that contained 185 open reading frames putatively identified as antimicrobial resistance genes. Of note, two MAGs were identified by GTDB-Tk as belonging to the “*Candidatus* Gracilibacteria” and “*Candidatus* Patescibacteria” lineages (12). Others identified included an unclassified *Dongiaceae* MAG that was previously associated with a wastewater treatment facility (13). A more detailed list of assembly statistics and taxonomy of all MAGs can be found in Table 1. As reported previously, no metagenomic sequence and therefore no MAGs were identified after barrier filtration within the system due to a lack of sufficient extractable or amplifiable DNA (4). Future work will further describe the ARGs and will identify non-ARG-containing MAGs and determine how they impact the operation of the OCWD AWPF.

**TABLE 1:** Detailed taxonomy, accession information, and assembly statistics of MAGs

Bin name	Taxonomy^*a*^	GenBank assembly accession no.	Total length (bp)	*N*_50_ (bp)	GC content (%)	Completeness (%)	Redundancy (%)
Bin_1_1	*Mycobacterium* sp. AWPF1	SSGB00000000	2,707,225	10,968	68.44	38.85	0.72
Bin_1_3	*Mycobacterium* sp. AWPF2	SSGA00000000	4,217,119	13,813	68.81	60.43	3.6
Bin_1_5	Unclassified *Actinobacteria* family UBA10799 bacterium AWPF1	SSET00000000	3,552,223	35,771	69.31	84.17	8.63
Bin_1_8	Unclassified *Actinobacteria* family UBA10799 bacterium AWPF2	SSES00000000	1,125,909	10,906	67.03	22.3	9.35
Bin_10_1	*Polynucleobacter* sp.	SSFP00000000	1,928,064	15,906	42.88	78.42	7.19
Bin_11_1	*Tolumonas* sp.	SSEX00000000	1,832,732	10,968	47.81	53.24	2.88
Bin_11_4	Unclassified *Cyclobacteriaceae* bacterium	SSEN00000000	2,496,402	8,305	45.51	36.69	9.35
Bin_11_5	*Cyclobacteriaceae* ELB16-189 sp.	VIGN00000000	1,066,491	8,282	43.86	36.69	12.23
Bin_12_3	Unclassified *Hyphomicrobiaceae* bacterium	SSEH00000000	2,999,406	8,617	55.82	7.19	12.23
Bin_14_1	*Novosphingobium* sp.	SSFU00000000	1,517,683	8,392	64.2	29.5	0.72
Bin_14_3	Unclassified *Gammaproteobacteria* bacterium AWPF1	SSEJ00000000	2,472,121	8,119	63.57	51.08	14.39
Bin_14_4	*Pseudoxanthomonas* sp.	SSFJ00000000	1,848,156	8,064	67.96	34.53	0
Bin_14_5	Unclassified *Gammaproteobacteria* bacterium AWPF2	SSEI00000000	1,859,286	10,791	62.7	43.17	41.73
Bin_15_1	*Nitrosomonas* sp. AWPF2	SSFW00000000	2,557,120	18,443	43.85	83.45	1.44
Bin_15_2	*Burkholderiaceae* UBA7693 sp.	SSGW00000000	2,650,165	9,223	49.03	22.3	0.72
Bin_15_3	*Moranbacterales* UBA1568 sp.	SSGE00000000	580,840	8,401	50.03	48.2	2.88
Bin_15_5	Unclassified *Moranbacterales* UBA1568 sp. AWPF1	SSEE00000000	880,789	8,806	45.6	66.91	19.42
Bin_15_6	Unclassified *Moranbacterales* UBA1568 sp. AWPF2	SSED00000000	1,461,468	7,107	48.36	52.52	15.83
Bin_17_3	Unclassified *Rhodocyclaceae* bacterium AWPF3	SSDZ00000000	3,614,409	8,707	60.2	60.43	10.79
Bin_19_1	*Ottowia* sp. AWPF1	SSFT00000000	2,464,482	7,364	68.69	17.27	2.16
Bin_19_2	*Ottowia* sp. AWPF2	SSFS00000000	2,330,319	8,499	69.66	42.45	6.47
Bin_19_5	*Thermomonas* sp.	SSFC00000000	1,187,954	11,860	67.14	38.13	0.72
Bin_2_1	Unclassified *Thermomicrobiales* family UBA6265 bacterium	SSDT00000000	2,680,321	10,114	60.7	49.64	7.91
Bin_20_1	*Niabella* sp.	SSFZ00000000	1,214,478	6,588	38.77	33.81	7.91
Bin_21_1	*Zoogloea* sp.	SSDR00000000	2,929,105	10,734	64.98	55.4	1.44
Bin_21_3	*Limnohabitans* sp. AWPF1	SSGJ00000000	1,670,846	9,983	63.22	19.42	0.72
Bin_23_2	*Flavobacterium* GCA_002422095.1	SSGM00000000	1,906,623	10,479	32.59	66.19	9.35
Bin_23_3	*Flavobacterium* sp.	SSGL00000000	2,075,117	12,249	32.14	33.09	2.88
Bin_24_1	*Rhodoferax* sp.	SSFF00000000	3,654,053	136,395	62.3	99.28	7.19
Bin_24_2	*Limnohabitans* sp. AWPF2	SSGI00000000	2,967,058	22,882	63.25	73.38	5.04
Bin_24_4	*Pseudorhodobacter* sp.	SSFK00000000	3,302,298	8,369	63.29	49.64	10.79
Bin_25_1	Unclassified *Rhodocyclaceae* bacterium AWPF1	SSDY00000000	2,178,082	10,805	64.25	62.59	2.16
Bin_25_2	*Flavobacteriales* PHOS-HE28 sp.	SSGN00000000	4,081,487	21,145	62.07	76.98	7.91
Bin_25_4	*Dechloromonas* sp.	SSGP00000000	3,029,215	19,114	62.22	58.99	5.76
Bin_26_1	Unclassified *Sphingomonadales* bacterium	SSDV00000000	3,053,326	60,967	64.22	99.28	0.72
Bin_26_2	*Mycobacterium mageritense*	SSGC00000000	7,650,754	60,194	66.88	79.14	5.76
Bin_26_3	Unclassified *Dongiaceae* bacterium	SSEL00000000	5,719,975	142,361	61.3	99.28	5.04
Bin_27_1	*Thauera aminoaromatica*	SSFD00000000	5,236,562	14,873	67.89	73.38	8.63
Bin_27_2	Unclassified *Burkholderiaceae* bacterium	SSER00000000	3,032,333	8,502	67.32	22.3	0.72
Bin_29_1	Unclassified *Mycobacterium* bacterium	SSEC00000000	6,629,733	18,228	66.91	85.61	7.91
Bin_29_2	*Mycobacterium arupense*	SSGD00000000	5,009,268	45,297	66.94	99.28	7.19
Bin_3_3	*Chryseobacterium cucumeris*	SSGV00000000	2,503,246	11,245	36.61	22.3	0
Bin_3_4	Unclassified *Chitinophagaceae* bacterium	SSEO00000000	1,501,328	17,275	33.51	50.36	0.72
Bin_3_8	Unclassified *Bacteroidia* bacterium	SSEU00000000	2,309,706	6,910	35.54	57.55	19.42
Bin_30	Unclassified *Burkholderiaceae* bacterium AWPF1	SSEQ00000000	6,171,595	8,294	70.29	47.48	4.32
Bin_31_2	*Aeromicrobium* sp.	SSHC00000000	1,861,791	18,902	58.23	82.73	1.44
Bin_34	Unclassified *Desulfurella* bacterium	SSEM00000000	6,288,683	10,284	58.83	3.6	5.04
Bin_35_1	*Pseudomonas monteilii*	SSFN00000000	5,048,709	26,274	63.26	51.8	2.16
Bin_35_2	*Thiobacillus* GCA_002343685.1	SSFB00000000	3,564,805	105,954	62.36	86.33	5.76
Bin_35_3	Unclassified *Nevskiaceae* bacterium AWPF1	SSEB00000000	3,246,569	241,139	65.99	86.33	2.16
Bin_36_2	*Crocinitomicaceae* UBA5422 sp. AWPF1	SSGS00000000	1,134,521	8,082	36.56	17.27	0
Bin_38_1	*Nitrosomonas* sp. AWPF1	SSFV00000000	2,613,687	22,274	44.56	57.55	0
Bin_39_1	*Pelomonas* sp.	SSFQ00000000	3,131,775	9,407	69.9	30.22	2.16
Bin_4_1	*Cupriavidus* sp.	SSGQ00000000	3,464,703	7,277	64.08	16.55	2.88
Bin_4_3	*Rhizobium* sp. AWPF1	SSFH00000000	4,147,977	10,631	61.05	18.71	1.44
Bin_42_2	*Methylophilus methylotrophus*	SSGG00000000	1,188,656	14,601	49.11	28.06	2.88
Bin_43_1	*Chryseobacterium* sp.	SSGU00000000	2,092,908	10,855	38.01	44.6	7.91
Bin_43_5	*Crocinitomicaceae* UBA5422 sp. AWPF2	SSGR00000000	641,979	9,434	42.11	35.25	17.27
Bin_44_1	*Betaproteobacteriales* UBA11063 sp. AWPF1	SSGY00000000	2,803,113	14,546	36.96	86.33	2.16
Bin_45_1	*Betaproteobacteriales* UBA11063 sp. AWPF2	SSGX00000000	2,467,391	70,519	35.22	79.14	1.44
Bin_45_4	“*Candidatus* Gracilibacteria” UBA5532 sp.	SSGK00000000	668,037	10,255	37.87	58.27	0.72
Bin_48_1	*Afipia* sp.	SSHB00000000	3,379,245	12,763	61.87	54.68	2.88
Bin_48_2	*Rhizobium* sp. AWPF2	SSFG00000000	2,353,640	12,778	60.82	38.13	0.72
Bin_5_1	*Lysobacter* sp.	SSGH00000000	4,387,597	162,300	65.61	94.24	5.76
Bin_5_5	Unclassified *Xanthomonadaceae* bacterium	SSEV00000000	3,535,438	16,226	63.31	47.48	0.72
Bin_5_7	Unclassified *Rhodocyclaceae* bacterium AWPF2	SSDX00000000	1,708,980	12,103	65.16	33.09	0.72
Bin_51_4	Unclassified *Saccharimonadales* UBA4665	SSDW00000000	818,540	13,215	50.23	61.87	8.63
Bin_52_1	*Pseudomonas alcaligenes*	SSFO00000000	3,682,067	12,539	64.72	71.22	3.6
Bin_52_2	*Pseudomonas* sp. AWPF1	SSFM00000000	1,540,019	11,651	61.81	17.27	4.32
Bin_54_1	*Nitrosomonas oligotropha*	SSFX00000000	2,754,397	36,184	49.22	67.63	0.72
Bin_54_3	*Nitrosomonas* GCA_002083595.1	SSFY00000000	2,292,023	8,445	48.32	29.5	6.47
Bin_55_1	46-32 GCA 001898405.1	SSHF00000000	5,043,020	214,644	44.27	94.96	0
Bin_55_2	*Methylophilus* sp.	SSGF00000000	2,747,675	94,685	50.49	66.91	2.16
Bin_56_1	*Thiothrix* sp.	SSFA00000000	4,155,808	145,664	44.77	69.78	3.6
Bin_56_2	*Pedobacter* sp.	SSFR00000000	2,341,987	206,071	38.94	98.56	0.72
Bin_57_1	*Rheinheimera* sp.	SSFI00000000	3,863,184	45,966	52.26	63.31	2.88
Bin_57_2	UBA7239 sp.	SSEW00000000	1,251,491	7,537	52.91	38.13	9.35
Bin_59_2	Unclassified *Nevskiaceae* bacterium AWPF2	SSEA00000000	2,177,696	27,134	59.28	27.34	5.04
Bin_63_2	Unclassified WS6 bacterium	SSDS00000000	1,242,531	11,680	44.02	34.53	9.35
Bin_64_3	*Romboutsia* sp.	SSFE00000000	381,413	7,806	27.61	45.32	0
Bin_65	*Crocinitomicaceae* 40-80 sp.	SSGT00000000	2,395,197	12,217	40.24	70.5	2.16
Bin_67	Unclassified *Spirochaetes* class UBA12135 bacterium	SSDU00000000	1,932,890	10,511	31.46	5.04	0
Bin_68_2	Unclassified *Moranbacterales* class UBA1568 bacterium	SSEF00000000	1,023,203	34,589	53.99	79.14	5.76
Bin_7_2	Unclassified *Burkholderiaceae* bacterium AWPF2	SSEP00000000	2,384,147	7,064	63.49	12.95	0.72
Bin_7_3	*Alicycliphilus* sp.	SSHA00000000	3,015,590	10,496	66.2	43.17	5.76
Bin_7_4	*Aquabacterium* sp. AWPF1	SSGZ00000000	1,352,035	7,819	65.11	21.58	0
Bin_7_5	*Pseudomonas* sp. AWPF2	SSFL00000000	1,978,447	9,650	63.83	51.08	10.79
Bin_7_6	*Aquabacterium* sp. AWPF2	VKOJ00000000	2,228,505	10,843	63.58	33.81	2.16
Bin_71	Unclassified *Leptospira* bacterium	SSEG00000000	1,795,065	38,625	54.29	6.17	0
Bin_8_1	*Acinetobacter* sp. AWPF1	SSHE00000000	2,045,996	23,569	41.75	75.54	2.16
Bin_8_2	*Thiothrix* sp. AWPF1	SSEZ00000000	1,300,697	7,394	44.99	30.22	1.44
Bin_8_3	*Thiothrix* sp. AWPF2	SSEY00000000	2,137,450	7,937	45.31	47.48	16.55
Bin_8_5	*Acinetobacter* sp. AWPF2	SSHD00000000	1,422,583	9,315	42.74	74.1	7.19
Bin_9_3	Unclassified *Elusimicrobia* order F11 bacterium	SSEK00000000	2,256,510	6,750	63.91	51.8	10.79
Bin_9_4	*Dokdonella* sp.	SSGO00000000	1,632,646	10,049	62.2	31.65	2.16

a Taxonomy given was identified using GTDB-tk and is the taxonomy reported within the NCBI accession record.

### Data availability.

Raw sequence reads are available under BioProject accession number PRJNA428383. Whole-genome sequences are available under the sequential BioSample accession numbers SAMN10026417 to SAMN10026511, which include annotations produced with the Prokaryotic Gene Annotation Pipeline (PGAP). Table 1 contains individual Web links to each bin assembly and annotation.
